# Controlling the behaviour of *Drosophila melanogaster* via smartphone optogenetics

**DOI:** 10.1038/s41598-020-74448-4

**Published:** 2020-10-19

**Authors:** Ilenia Meloni, Divya Sachidanandan, Andreas S. Thum, Robert J. Kittel, Caroline Murawski

**Affiliations:** 1 Kurt-Schwabe-Institut für Mess- und Sensortechnik Meinsberg e.V., Kurt-Schwabe-Str. 4, 04736 Waldheim, Germany; 2 Department of Animal Physiology, Institute of Biology, Universität Leipzig, Talstr. 33, 04103 Leipzig, Germany; 3 Carl-Ludwig-Institute for Physiology, Universität Leipzig, Liebigstraße 27, 04103 Leipzig, Germany; 4 Department of Genetics, Institute of Biology, Universität Leipzig, Talstr. 33, 04103 Leipzig, Germany

**Keywords:** Neuroscience, Optogenetics, Drosophila, Lasers, LEDs and light sources

## Abstract

Invertebrates such as *Drosophila melanogaster* have proven to be a valuable model organism for studies of the nervous system. In order to control neuronal activity, optogenetics has evolved as a powerful technique enabling non-invasive stimulation using light. This requires light sources that can deliver patterns of light with high temporal and spatial precision. Currently employed light sources for stimulation of small invertebrates, however, are either limited in spatial resolution or require sophisticated and bulky equipment. In this work, we used smartphone displays for optogenetic control of *Drosophila melanogaster*. We developed an open-source smartphone app that allows time-dependent display of light patterns and used this to activate and inhibit different neuronal populations in both larvae and adult flies. Characteristic behavioural responses were observed depending on the displayed colour and brightness and in agreement with the activation spectra and light sensitivity of the used channelrhodopsins. By displaying patterns of light, we constrained larval movement and were able to guide larvae on the display. Our method serves as a low-cost high-resolution testbench for optogenetic experiments using small invertebrate species and is particularly appealing to application in neuroscience teaching labs.

## Introduction

*Drosophila melanogaster* is a powerful genetic research organism to disentangle the relation between behaviour and brain structure, neuronal activity, and the molecular processes involved^1^. In order to control neuronal function, binary expression systems can be used to selectively target small populations of neurons or even individual cells^2,3^. A versatile method to control neuronal activity non-invasively and with high spatial and temporal precision is optogenetics. The technique is based on the genetic expression of light-sensitive ion channels in the cell membrane^4–6^, which enables activation and inhibition of neurons in response to light. Optogenetics has been well established to interrogate behaviour in *Drosophila melanogaster*^7^, with studies ranging from cardiac pacing^8^ over locomotion control^9^ to investigations of memory formation^10,11^.

The optical stimulation of free-roaming *Drosophila* requires light to be delivered over a large arena, which is typically achieved using LEDs^12–14^. Multiple LED panels or filters may be used to switch between different emission colours^15,16^. Targeting specific regions of interest in larvae or flies is possible using arrays of organic light-emitting diodes (OLEDs)^17^, projectors^18,19^, or focussed lasers^20,21^. Typical experimental setups used for light delivery, however, require custom hardware in order to enable time control, are expensive, provide only a single colour for stimulation, or achieve only limited spatial resolution. To overcome these drawbacks, we suggest using smartphone displays for optogenetic stimulation. This combines simplicity and low cost with the potential to achieve high spatiotemporal resolution by projecting light patterns of different colours.

Smartphones are highly attractive as sensing and controlling devices and have found several applications in a biological context, e.g., as portable microscopes^22–24^, for tracking of small animals^25^, or as wireless communication system to control light sources for optogenetics^26–28^. So far, however, smartphones were mainly used as a passive device for sensing and data recording, processing, and transmission. Here, we use the smartphone for active control of neuronal function in *Drosophila* larvae and flies. For this purpose, we developed an Android-based smartphone app that allows spatiotemporal and spectral control over light output from the display and used this to stimulate several sets of neurons expressing different channelrhodopsins. Moreover, we demonstrate the possibility to spatially control larval movement by providing fine patterns of light. Our research proves that smartphone displays can be employed as a low-cost, high-resolution testbench for optogenetic interrogation of neurons in *Drosophila*.

## Results and discussion

### Display characterisation

Optogenetic light sources for stimulation of *Drosophila melanogaster* larvae have to fulfil certain requirements: (1) The emission spectrum of the light source needs to overlap with the activation spectrum of the selected light-sensitive protein. (2) Depending on the application, spatial resolution in the range between 10 µm and 10 mm is required: optical dissection of neural circuits in the CNS^29^ and targeting of distinct synaptic boutons at the neuromuscular junction^30^ require a resolution in the order of 10 µm; stimulation of individual larval abdominal segments is achieved with light structured to around 100 µm^17^; optogenetic olfactory preferences and associated learning may be studied at resolutions on the centimetre scale^31^. (3) High temporal resolution is required. While switching times of around 100 ms will be sufficient for most behavioural experiments^32^, faster switching may be required, e.g., for optogenetic pacing^8^. (4) The power density of the light source needs to be high enough to achieve neuronal activation. Here, the wild-type Channelrhodopsin-2 (ChR2) typically requires light intensities in the order of mW/mm^2^. However, more light-sensitive ChRs that respond to power densities from 1 µW/mm^2^ or even less have been developed in recent years^11,33–35^. Such efficient photostimulation is important to avoid unspecific effects, e.g., due to excessive heat generation.

**Figure 1:**
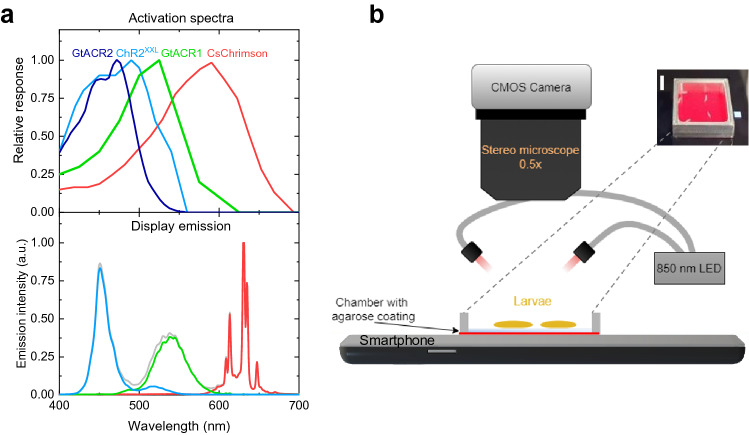
Smartphone display for optogenetics. (**a**) Activation spectra of light-sensitive ion channels (top)^11,33,34^ and emission spectra of Honor 8 smartphone display used (bottom); lines in emission spectra correspond to display colour, given by RGB values: (0,0,255; blue), (0,255,0; green), (255,0,0; red), and (255,255,255; white). (**b**) Sketch of the experimental setup. Inset: photograph of the chamber containing several larvae, illuminated by red light from the smartphone. Scale bar: 5 mm.

For our study, we selected four different highly efficient ChRs: The cation-conducting CsChrimson and ChR2^XXL^ for neuronal activation and the anion-conducting GtACR1 and GtACR2 for neuronal silencing. The response of optogenetic reporters depends on the light-sensitive ion-channel used, in which cells they are expressed, the developmental stage of the animal, and the spectral overlap between the light source and the ChR. Furthermore, sensitivity is strongly influenced by the amount of supplemented all-*trans* retinal (ATR), a light-isomerizable chromophore required to induce the opening of the transmembrane protein^36^, although ChR2^XXL^ is also effective without addition of ATR^11^. Figure 1a shows the activation spectra of the four ChRs used. CsChrimson is a red-shifted ChR with strong light sensitivity from 510 to 640 nm and residual sensitivity to blue light. Response in *Drosophila* larvae was reported to brightness levels of less than 5 µW/mm^2^^37^. ChR2^XXL^ is the most efficient ChR reported so far with activation achieved at 0.04 µW/mm^2^ and a spectrum peaking at 460 nm^11^. GtACR1 is sensitive to green and blue light using illumination of 2 µW/mm^2^ or less, while GtACR2 is only sensitive to blue light and requires higher brightness levels of around 5 µW/mm^2^^35,38^.

To investigate whether smartphone displays fulfil the above-mentioned requirements, we measured the emission characteristics of ten commercial smartphones and of one tablet. Current smartphone displays are either based on active matrix OLED or on liquid crystal display (LCD) technology. While OLED displays control the light emission of each pixel individually, LCDs require backlight illumination leading to residual emission when the display is black. Emission spectra of red, green, and blue (RGB) sub-pixels of OLED and LCD displays show only small differences in peak positions (Supplementary Fig. S1). For our following optogenetic experiments, we used an Honor 8 smartphone containing an LCD display. The action spectra of ChR2^XXL^ and GtACR2 show good spectral overlap with the emission from blue sub-pixels (Fig. 1a); GtACR1 overlaps well with green and blue and CsChrimson with green and red sub-pixels.

The spatial resolution of an area emitter depends on the size of the light-emitting area, the distance between emitter and observer, and the angular distribution of the light emission. Sub-pixel dimensions of the Honor 8 smartphone are 41 µm × 12 µm. The thickness of the cover glass contributes largely to the distance between light source and stimulated cells and is in a range of 0.6 to 1.2 mm (Supplementary Table S1; 680 µm for Honor 8). The angular distribution of area emitters generally follows a cosine-distribution (Lambertian emission). However, the use of polarizers and optical filters often causes preferential emission into forward direction. We measured the angular characteristics of the Honor 8 display using a goniometer and found substantial shaping of emission into forward direction (Supplementary Fig. S2). Using simple geometrical considerations and taking the angular distribution and glass thickness of the Honor 8 into account, we estimated the lateral spread of light at the top of the glass thickness. At a 95% confidence interval, the light emitted by a single pixel spreads by 665 µm compared to the pixel centre. For the other smartphones, we estimated a maximum light spread of 2.5 mm assuming Lambertian emission. However, we expect that with preferential forward emission most modern phones will achieve a spatial resolution of 1 mm or better.

Typical smartphone displays provide refresh rates of 60 Hz or even higher and thus should enable sufficient temporal control for neuronal responses. We measured the rise and fall times of the Honor 8 smartphone, which were 13 ms and 21 ms, respectively.

Finally, we measured the power density of the smartphone and tablet displays for each sub-pixel using a calibrated photodiode. Depending on the smartphone and the sub-pixel colour, between 0.8 and 2.8 µW/mm^2^ were detected (Supplementary Table S1). The Honor 8 display showed power densities of 1.8 µW/mm^2^ for blue and 2.1 µW/mm^2^ for both green and red sub-pixels, leading to a measured total intensity of 6.6 µW/mm^2^ for white light. Considering the light intensity requirements of the ChRs used in our work, we expected to elicit neuronal responses with CsChrimson, ChR2^XXL^, and GtACR1, while light intensity may not suffice for activation of GtACR2.

In order to control the spatiotemporal light output of the smartphone display, we developed an Android-based application and made this available open source. The app allows the user to define a temporal sequence of rectangular or circularly shaped light patterns of adjustable size, colour, intensity, position, speed, and moving direction. Furthermore, it is possible to input a picture as light pattern in order to display complex shapes for a specific period of time.

### Spectral dependency of activation and inhibition

In the following, we investigated whether the smartphone delivers sufficient spectral overlap and light intensity to achieve neuronal activation and inhibition in *Drosophila* larvae expressing the ChRs introduced above. Figure 1b shows our experimental setup. Larvae were placed on a 1 mm thin agarose sheet that is located inside an aluminium chamber positioned on the Honor 8 smartphone display. Larval behaviour was recorded underneath a stereo microscope with a CMOS camera under infrared illumination (850 nm).

**Figure 2:**
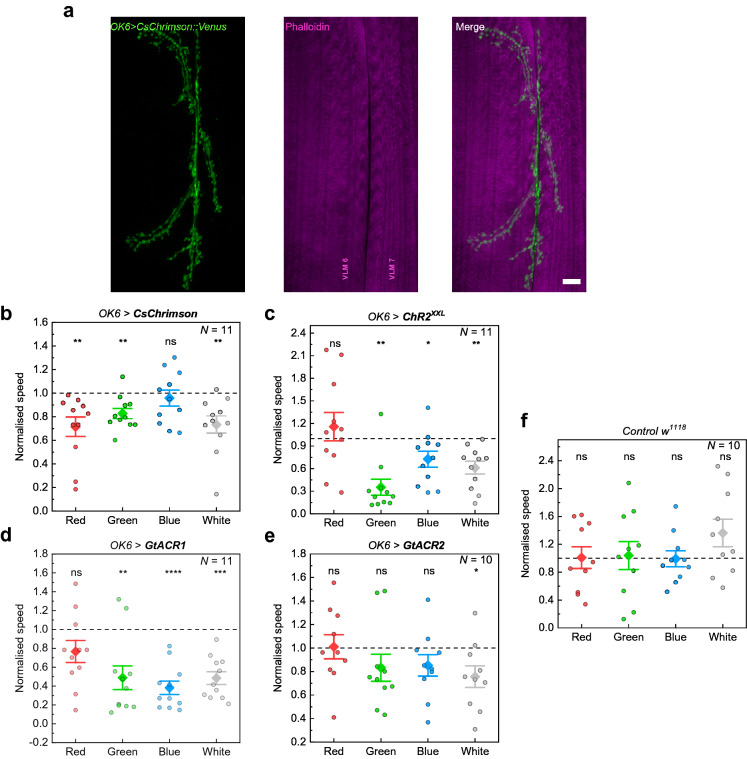
Behavioural response of larvae expressing different light-sensitive ion channels in motor neurons (*OK6-GAL4*) to optogenetic stimulation with the Honor 8 smartphone display. (**a**) Motoneuronal expression of Chrimson::Venus obtained by antibody staining against the photoprotein (green). Phalloidin stained ventral longitudinal muscles (VLM) depicted in magenta. Scale bar: 10 µm. (**b**)–(**f**) Larval responses to illumination with different colours in temporal sequences of red (R), green (G), blue (B), and white (W) light with intermediate black periods (Supplementary Fig. S3). Data show the speed of the larvae normalised to the previous black period as obtained by tracking the head (see Methods). (**b**) Neuronal activation of *OK6* > *CsChrimson* larvae. Stimulation period of 20 s with 60 s of rest (black period). (**c**) Neuronal activation using *OK6* > *ChR2*^*XXL*^. Stimulation period: 10 s; black period: 150 s. (**d**) Neuronal silencing using *OK6* > *GtACR1*. Stimulation period: 20 s; black period: 60 s. (**e**) Neuronal silencing using *OK6* > *GtACR2*. Stimulation period: 30 s; black period: 60 s*.* (**f**) Normalised speed of *w*^*1118*^ control larvae. Stimulation period: 20 s; black period: 30 s. *N*: number of larvae; whiskers: s.e.m.; diamonds: mean. Significance calculated via one-sample two-tailed t-test: ns: not significant (*p* > 0.05), *p < 0.05, **p < 0.01, ***p < 0.001, ****p < 0.0001.

We delivered a light sequence to larvae starting with a black display (residual light intensity of 5.3 × 10^–3^ µW/mm^2^) followed by red, green, blue, and white light stimulation, each interrupted by a black period for larval relaxation (Supplementary Fig. S3). The time of stimulation and black period were adjusted for each experiment taking into account the dynamics of the ChR used and are given in the figure captions. We expressed ChRs using the GAL4/UAS-system in motor neurons with *OK6-GAL4*^39^ (Fig. 2a). Both activation and inhibition of motor neurons evoke behavioural changes, i.e., muscle contraction and immobilisation in the case of activation^11^, and muscle relaxation, again leading to immobilisation, for inhibition^29^. We used third instar larvae for all experiments and raised flies on food supplemented with 0.5 mM ATR.

Figure 2b–e shows the speed of larvae during each stimulation period for all ChRs investigated, normalised to the speed of the larvae during the black period preceding the stimulation. CsChrimson-expressing larvae showed a significant decrease in speed when stimulated with green, red and white light (Fig. 2b), which is in agreement with the activation spectrum of CsChrimson (Fig. 1a). We observed simultaneous contraction of all muscles through activation of the motor neurons, which considerably disturbed coordinated crawling. A similar response was observed when stimulating ChR2^XXL^-expressing larvae (Fig. 2c) with green, blue, and white light, in agreement with the activation spectrum of ChR2^XXL^. Since ChR2^XXL^ ion channels close very slowly, we extended black periods to 150 s (60 s in all other cases).

Expressing the light-gated inhibitors GtACR1 and GtACR2 in motor neurons, we observed cessation of larval crawling due to muscle relaxation in response to blue, green, and white light for GtACR1 (Fig. 2d, Supplementary Video S1). The light-induced response of larvae expressing GtACR2 was weaker compared to GtACR1 and a significant effect was only observed for stimulation with white light (Fig. 2e). This result is in line with expectations since the power density required to activate GtACR2 is substantially higher compared to GtACR1^35,38^. Although GtACR2 is mainly activated by blue light, small spectral overlap exists also to green light emitted by the smartphone (Fig. 1a). Thus, the added intensity of green and blue light was sufficient to inhibit the larval motor system. Light stimulation of *w*^*1118*^ control larvae, raised under the same conditions as ChR-expressing larvae, showed no alteration in speed (Fig. 2f).

The response observed for each ChR is in agreement with the spectral overlap of display emission and activation spectrum of the ChR. Both activation and inhibition of neuronal activity have been achieved and the light intensity of approximately 2 µW/mm^2^ provided by each display colour was sufficient to robustly control larvae expressing CsChrimson, ChR2^XXL^, and GtACR1.

### Targeting different sets of neurons

One of the big challenges in optogenetics is to address the deep-seated neurons, where scattering inside the tissue spreads the light and decreases the power density. Hence, such neurons usually require higher light intensities or longer wavelengths^40–42^. In the following, we extended our previous studies on motor neurons to the activation of muscles using the *G7-GAL4* driver^43^, stimulation of all sensory neurons (*5-40-GAL4*)^44,45^, and activation of class IV multidendritic neurons (*ppk-GAL4*)^46^ in order to show that smartphones can be applied more universally to control different cells. For all drivers, we used the reporters *UAS-CsChrimson* and *UAS-ChR2*^*XXL*^ and applied a similar stimulation sequence as before.

**Figure 3:**
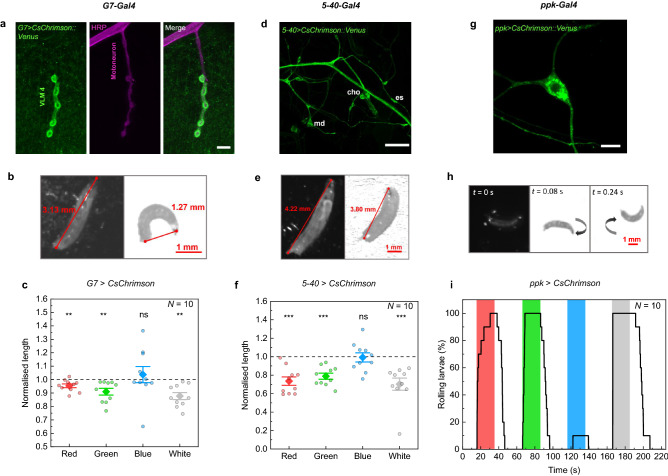
Optogenetic activation of muscles (*G7-GAL4*, (**a**)–(**c**)), several sensory neurons (*5-40-GAL4*, (**d**)–(**f**)), and class IV multidendritic neurons (*ppk-GAL4*, (**g**)–(**i**)) by expression of CsChrimson. (**a**) Expression of *CsChrimson::Venus* by *G7-GAL4* as observed by antibody staining (green). Magenta represents the motor neuron stained with anti-HRP (horseradish peroxidase), a neuronal membrane marker. Scale bar: 10 µm. (**b**)–(**c**) Stimulation of muscles in larvae expressing *G7* > *CsChrimson*. (**b**) Representative images from video recordings before (left) and after (right) illumination with white light. (**c**) Normalised larval length for a sequence of 20 s stimulation for each colour and 60 s black period. (**d**) *5-40-GAL4* drives expression of *Chrimson::Venus* in the peripheral nervous system consisting of chordotonal (cho) neurons, external sensory (es) neurons, and multidendritic (md) neurons. Scale bar: 50 µm. (**e**)–(**f**) Stimulation of all sensory neurons in larvae expressing *5-40* > *CsChrimson*. Stimulation period: 20 s; black period: 30 s. (**g**) Using *ppk-GAL4*, *Chrimson::Venus* is exclusively expressed in class IV dendritic arborisation (C4da) neurons. Scale bar: 10 µm. (**h**)–(**i**) Stimulation of larvae expressing *ppk* > *CsChrimson*. (**h**) Image sequence before (*t* = 0 s) and after stimulation with white light displaying a characteristic rolling behaviour. (**i**) Percentage of larvae that were rolling over time. Stimulation period: 20 s; black period: 40 s. *N*: number of larvae; whiskers: s.e.m.; diamonds: mean. Significance calculated via one-sample two-tailed t-test: ns: not significant (*p* > 0.05), **p < 0.01, ***p < 0.001.

While *OK6-GAL4* drives expression in presynaptic motor neurons, we now controlled the motor system postsynaptically by targeting muscles using *G7-GAL4* (Fig. 3a). Optogenetic stimulation of CsChrimson-expressing larvae using the smartphone caused larvae to contract, roll onto their sides, and eventually remain immobilized in a curled C-shape position (Fig. 3b, Supplementary Video S2). We quantified this response by measuring the mean normalised length of larvae during each stimulation period and observed a significant decrease of the length upon red, green, and white light stimulation (Fig. 3c). We repeated the experiment with *G7* > *ChR2*^*XXL*^ larvae and obtained the same behavioural response upon stimulation with green, blue and white light (Supplementary Fig. S4a). The evoked contraction also affected larval speed, which decreased during the same stimulation periods (Supplementary Fig. S4b). Furthermore, no response was observed in *w*^*1118*^ control larvae analysing the mean normalised length and speed (Supplementary Fig. S5, Fig. 2f).

Next, we expressed CsChrimson in all sensory neurons using the *5-40-GAL4* driver. Figure 3d shows expression throughout the peripheral nervous system including chordotonal neurons, external sensory neurons, and multidendritic neurons. Neuronal activation using this driver line caused a full-body contraction resulting in immobilization and a reduced length of the larvae (Fig. 3e), as described previously^17,44,45,47^. In agreement with the action spectrum of CsChrimson, we achieved activation in response to green, red, and white light (Fig. 3f). The same behavioural response was obtained in *5-40* > *ChR2*^*XXL*^ larvae but using blue, green, and white light stimulation (Supplementary Fig. S4c).

Finally, we performed experiments with larvae expressing CsChrimson in class IV dendritic arborisation (C4da) neurons using *ppk-GAL4* (Fig. 3g). Activation of *ppk* (*pickpocket*)-expressing neurons elicits nociceptive behaviour, characterized by a cork-screw body roll^46^. Again, we stimulated larvae with a colour sequence but analysed the data by counting the number of rolling larvae (Fig. 3h,i). All larvae started rolling within the 20 s stimulation in response to red, green and white light. The rolling behaviour is accompanied by an increase in speed (Supplementary Fig. S6). We also expressed ChR2^XXL^ in C4da neurons using *ppk-GAL4* and observed similar rolling behaviour with corresponding speed increase for stimulation with blue and white light (Supplementary Video S3, Supplementary Fig. S4d,e). ChR2^XXL^, however, led to prolonged photoactivation where the larvae continued rolling for around 3 min after white light stimulation. This can be attributed to the extended open-state lifetime of ChR2^XXL^^11^.

**Figure 4:**
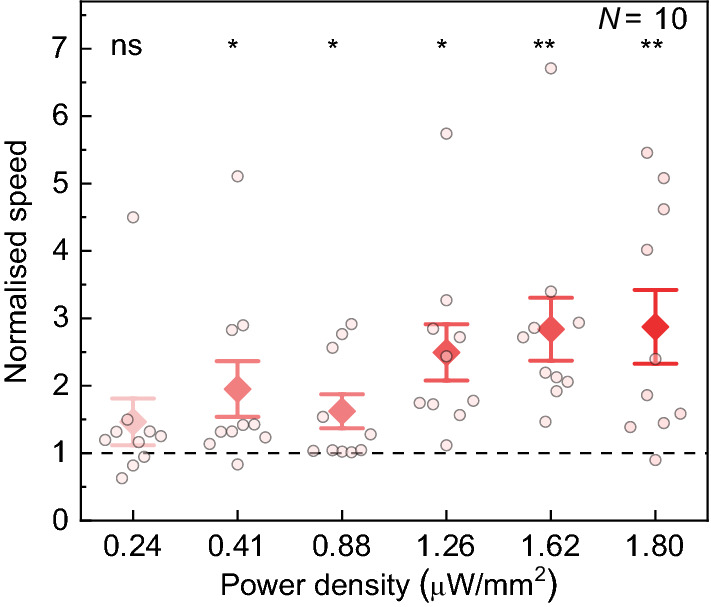
Influence of display brightness on normalised speed of *ppk* > *CsChrimson* larvae for illumination with red light of increasing power density. Stimulation period: 20 s; black period: 30 s. *N*: number of larvae; whiskers: s.e.m.; diamonds: mean. Significance calculated via one-sample two-tailed t-test: ns: not significant (*p* > 0.05), *p < 0.05, **p < 0.01.

Since the power density delivered by different smartphone displays may vary (with a minimum of 0.8 µW/mm^2^ measured for blue emission from the Fairphone 2; Supplementary Table S1), we measured the dose–response curve exemplarily for *ppk* > *CsChrimson* larvae. The power density was adjusted by changing the RGB value [note that there is no linear relationship between RGB-values and power density (Supplementary Fig. S7)]. Figure 4 presents the normalised speed of larvae when stimulated with red light of increasing intensity. The minimum power density required to evoke optogenetic activation was 0.41 µW/mm^2^. Thus, we expect that modern smartphone models should generally be capable of stimulating a response. However, the significant increase in speed change with increasing brightness suggests that smartphones with displays delivering high power density will be advantageous.

### Spatial control of larval movement

An important advantage of smartphone displays over traditional light sources is the possibility to deliver light with high spatial resolution. This makes it possible to perform dynamic experiments with *Drosophila,* which are currently difficult to achieve^31^. Here, we investigate the possibility to control larval movement by displaying spatiotemporal light patterns on the smartphone display. For this, we confined *ppk* > *CsChrimson* larvae in two different geometric shapes. Since activation of C4da neurons is nociceptive and evokes avoidance, this enabled us to design experiments in which light is used to imprison larvae.

**Figure 5:**
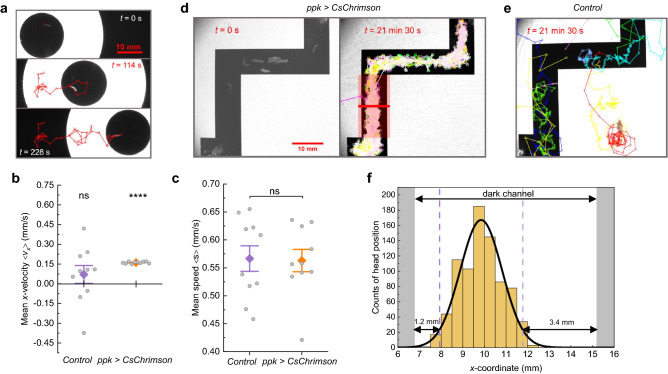
Spatial control of larval movement by patterned illumination with a smartphone. (**a**) Guidance of free-behaving *ppk* > *CsChrimson* larvae. Images show representative frames from Video S4. The larva is enclosed within a ring of white light that moves at a speed of 0.17 mm/s in *x*-direction. The red track shows the larval trajectory. (**b**) Mean velocity < *v*_x_ > in *x*-direction and (**c**) mean speed < *s* > of 10 *w*^*1118*^ control larvae and 10 *ppk* > *CsChrimson* larvae confined in the conditions described in (**a**). Whiskers: s.e.m.; diamonds: mean. Significance calculated via one-sample two-tailed t-test (in (**b**)) and two-sample two-tailed t-test (in (**c**)): ns: not significant (*p* > 0.05), ****p < 0.0001. (**d**) Confinement of 11 *ppk* > *CsChrimson* larvae inside a black maze surrounded by white light (Supplementary Video S6). Left: Image of larvae positioned inside the maze. Right: Tracks of 11 larvae over the course of 21 min 30 s. (**e**) Tracks of 10 *w*^*1118*^ control larvae over the course of 21 min 30 s (Supplementary Video S7). (**f**) Histogram and Gaussian fit of the larval head positions along the red line (20 mm width) shown in (**d**). Dashed lines mark the 95% confidence interval.

The first experiment consists of a ring of light (inner diameter: 17 mm, outer diameter: 47 mm) moving horizontally on the display at a speed of 0.17 mm/s, similar to previous experiments that used a focussed projector as a light source and the *painless* promoter to drive ChR2 expression in nociceptive neurons^48^. Figure 5a shows representative time-lapse images of a larva constrained inside the inner circle (black/safe zone; see Supplementary Video S4). Larvae moved freely inside the safe zone but performed turns when approaching the ring of light. By moving the ring, larvae could be guided to different places on the display. In contrast, *w*^*1118*^ control larvae regularly moved out of the ring (Supplementary Video S5). In order to quantify this behaviour, we tracked larval positions and measured the velocity in *x*-direction and speed for *w*^*1118*^ and *ppk* > *CsChrimson* larvae (Fig. 5b,c). While the speed of both groups is very similar, the velocity graph shows significant differences: *ppk* > *CsChrimson* larvae were constrained to follow the ring in *x*-direction resulting in a mean ± s.e.m. *x*-velocity of 0.161 ± 0.002 mm/s, which is comparable to 0.17 mm/s of ring velocity. Furthermore, we calculated a prisoner index for each larva, which is defined as time spent inside the ring divided by the total time, and obtained 1.00 ± 0.00 (mean ± s.e.m.) for *ppk* > *CsChrimson* larvae and 0.29 ± 0.04 for controls (*N* = 10 larvae in each case).

In our second experiment, we confined *ppk* > *CsChrimson* larvae inside an optical maze that consists of a black ‘tunnel’ surrounded by white light (Fig. 5d). We placed 11 larvae inside the maze and tracked the movement of the head of each larva for 21 min 30 s. As a result, 10 larvae stayed inside the maze for the whole time (prisoner index: 0.91 ± 0.09 (mean ± s.e.m.); Supplementary Video S6), while for *w*^*1118*^ control larvae only 2 out of 10 remained inside the maze (0.36 ± 0.13; Fig. 5e; Supplementary Video S7). While some control larvae stayed inside the maze due to negative phototaxis, the power density used in our experiments is six times lower compared to earlier studies on *wild-type* animals^49^. Comparing the area ratio of the maze (black zone) to the rest of the chamber, a prisoner index of 0.22 would be expected if controls were completely unaffected. While some weak phototaxis may have contributed to the increased prisoner index observed, further bias may have arisen from the initial positioning of larvae inside the maze as opposed to a uniform distribution of larvae inside the whole chamber.

In order to characterise the resolution of our setup, we examined the distribution of *ppk* > *CsChrimson* larvae inside the maze. While the light distribution depends strongly on the distance between the light source and the animals, i.e., the thickness of the cover glass on top of the display and the agarose sheet, the light sensitivity of the larvae will also play a role. We tracked the head position of all larvae in the maze over time and calculated a histogram of the *x*-position within the vertical channel of the maze (see the red marked area in the right panel of Fig. 5d). The histogram of the distribution visualises the distance that larvae kept towards the edges of the maze (Fig. 5f). Assuming a Gaussian distribution, 95% of the larvae remained inside the central part of the channel, 1.2 mm and 3.4 mm away from the left and right borders, respectively. We attribute the difference observed between left and right side to the different width of the light-emitting areas, which are 7 mm wide at the left side of the channel and 37 mm wide at the right side. This may have caused a difference in the distribution of light inside the channel thus leading to the observed distribution. While further analysis will be needed to reveal the mechanisms in detail, we can conclude that high spatial resolution has been achieved suitable to confine and guide larvae.

## Conclusions

We have demonstrated that smartphone displays are capable of controlling optogenetic activation and silencing in *Drosophila* larvae. For this, we developed an Android-based app that allows high spatiotemporal control over the emitted light. Larval response correlated with the activation spectrum of the selected optogenetic tool and we achieved successful stimulation of larvae expressing CsChrimson, ChR2^XXL^, and GtACR1 in different sets of cells. Furthermore, we demonstrated the possibility to use static and dynamic light patterns to constrain larval movement. Smartphone-based optogenetics enables powerful technical improvements for understanding how molecular and cellular processes are embedded in neuronal circuits to bring about fundamental properties of behaviour and basic forms of cognition — such as taxes, kineses, and associative memory.

Smartphone optogenetics is not limited to controlling *Drosophila* larvae, but has also great potential for application in adults. In a preliminary experiment, we already explored the possibility to activate neurons in *Drosophila* flies using a simplified experimental setup and achieved similar responses as in larvae (Supplementary Fig. S8, Supplementary Video S8). We believe that smartphone optogenetics will also be interesting for studying other small species such as *Chlamydomonas reinhardtii*^50^, *C. elegans*^51^, and *Zebrafish*^52^. For stimulation of larger animals or bigger cultures, tablet displays may be used instead of smartphones to deliver light to larger arenas.

Besides the use of our smartphone-based light source in research labs, the simplicity of the setup and ubiquitous availability of smartphones also makes it ideal for application in teaching labs and outreach activities. In a first trial, we have implemented a smartphone-based optogenetics activity in a neuroscience lab course at Leipzig University, which confirmed that students could replicate results from Figs. 3i and 5a using other smartphone models (Xiaomi Mi 4i, Sony Xperia Z5, and Motorola Moto G). Activities may include, e.g., investigation of the spectral sensitivity of different channelrhodopsins, learning how specific sets of neurons control larval behaviour, or include learning and memory tasks. We believe that the interdisciplinary character and the possibility to test own research hypotheses will increase the learning experience of students. To conclude, smartphone optogenetics is highly versatile and ready to use in both research and teaching environments.

## Methods

### Smartphone display characterisation

Pixel sizes were measured through a Stereo Microscope (Nikon SMZ25) coupled to an sCMOS-camera (Andor Zyla 4.2 PLUS). The thickness of the cover glass on top of the display was measured with the same setup by focussing either on the pixel or on the top of the display. Spectra were acquired with a calibrated spectrometer (AvaSpec-ULS2048CL-EVO). Activation spectra of Channelrhodopsins used were extracted from the original publications using the tool WebPlotDigitizer. The power density of the emitted light was measured with a calibrated photodiode (Thorlabs PDA100A2) coupled to a Multimeter (Keithley DMM6500). The angular distribution of the emitted spectrum was recorded using a home-built goniometer, composed of the spectrometer and a rotation stage (Thorlabs K10CR1/M), and controlled by a python script. The temporal resolution of the display was measured with the photodiode connected to an oscilloscope (Tektronix MDO3054). Rise and fall times are given as thresholds exceeding 90%/falling below 10% of intensity.

### *Drosophila* strains and fly husbandry

The following fly strains were used: *5-40-GAL4*^45^, *OK6-GAL4*^39^, *G7-GAL4*^43^ (gift from A. DiAntonio), *ppk-GAL4* (BDSC#32078), *UAS-CsChrimson* (BDSC#55136), *UAS-ChR2*^*XXL*^ (BDSC#58374)^11^, *UAS-GtACR1*^53^, *UAS-GtACR2*^38^, and *w*^*1118*^. All fly crosses were raised in the dark at 25 °C on conventional cornmeal-agar medium supplemented with 0.5 mM all-*trans*-retinal (ATR). Control *w*^*1118*^ flies were also grown on 0.5 mM ATR-supplemented food.

### Immunostaining and confocal microscopy

Larval fillet preparation, subsequent immunostaining and confocal microscopy were performed as described in Ehmann et al.^54^. Briefly, wandering third instar larvae were dissected using ice cold HL-3^55^, followed by fixation with 4% paraformaldehyde for 10 min at room temperature. Blocking was performed using 5% normal goat serum in 0.05% PBST (phosphate-buffered saline containing 0.05% Triton-X) for 30 min at room temperature, followed by primary staining with mouse anti-GFP antibody (1:500; Sigma-Aldrich, SAB4200681; RRID:AB_2827519) in 0.05% PBST at 4 °C, overnight. The next day, the fillets were washed 6× for 10 min each and incubated in secondary antibody Alexa Fluor-488-conjugated goat-α-mouse (1:250; Invitrogen, A-11001; RRID:AB_2534069). For *G7-GAL4* and *OK6-GAL4* expression studies, Cy3 conjugated anti-Horseradish Peroxidase (HRP) (1:250; Jackson ImmunoResearch Labs 123-165-021, RRID:AB_2338959) and Phalloidin-532 (1:250; Thermo Fisher Scientific A22282, RRID:AB_2716808) were used as counterstains, respectively. Secondary antibody staining was done for 2 h at room temperature. After washing, the fillets were stored in Vectashield at 4 °C overnight before being mounted for confocal microscopy. The confocal images were acquired using a Zeiss LSM 800 microscope with Plan-Neofluor 16×/0.50 or Plan-Apochromat 63×/1.4 oil immersion objectives depending on the region of interest.

### Optogenetic imaging setup

All optogenetic measurements with *Drosophila* larvae were recorded underneath a stereomicroscope (Nikon SMZ25, P2-SHR Plan Apo 0.5×/0.075 NA objective) with an sCMOS camera (Andor Zyla 4.2 PLUS). A 645 nm long pass filter was mounted in front of the camera to avoid overexposure due to the display illumination. Using this filter, only light from the red sub-pixels of the display was detected by the camera. Larvae were imaged under infrared light using an LED light source (Thorlabs M850L3; 850 nm peak, 30 nm FWHM) projected onto the sample via a dichroic mirror (805 nm cutoff wavelength) and a Y-branched fibre bundle, causing a light intensity of approximately 0.25 mW/mm^2^ at the sample. Videos were acquired from the sCMOS camera using Nikon NIS-Elements software in .avi format at a frame rate of 20 Hz.

For optogenetics experiments, third instar larvae were taken out of the vials in dim red light (dim blue light for CsChrimson-expressing flies), gently washed in DI water, and placed on a thin sheet of agarose (1.5% w/v solution in DI water) located on top of the smartphone display. All optogenetic stimulation experiments were performed using an Honor 8 smartphone controlled through a custom Android app.

Experiments with adults were recorded with a smartphone camera (Samsung Galaxy S10+) at an acquisition rate of 25 Hz in the dark. Flies were placed inside an aluminium box containing 4 channels, which were covered on top and bottom sides with a glass slide, allowing to see through the box. In each channel, 3 flies were carefully placed using a brush. The box was then mounted vertically in front of the upright standing Honor 8 display used for optogenetic stimulation.

Before starting optogenetic experiments, animals got accustomed to their new environment for approximately 10 min in the dark. If not specified differently, larvae and flies were stimulated with a sequence of red, green, blue and white light, each defined by their respective RGB values set to highest intensity (255). Each stimulation was followed by a period of rest; in this period, the smartphone display was black. In order to synchronise camera recordings with the timed illumination from the smartphone display, a small spot of white light was displayed outside the experimental area whenever the display illumination was turned on.

### Data analysis

Larval speed was measured by tracking the head positions manually every second (every 5 s for the maze experiment) in ImageJ using the plugin MTrackJ. Larval length was analysed by tracking both head and tail positions, and calculating the geometric distance of the two points. Subsequently, the length and speed were normalised to the mean value measured in the resting period preceding the stimulation for each larva. Speed and length were calculated as mean values for the duration of the whole period, except for *OK6* > *ChR2*^*XXL*^, and *5-40* > *ChR2*^*XXL*^, since full recovery was achieved in those experiments only shortly before the next stimulation period started. Here, the speed/length was calculated from the last 2 s/1 s before the start of the next stimulation period. The fraction of rolling *ppk* larvae was analysed visually for every frame. Statistics and significance via two-tailed t-test were performed using OriginPro. All *p* values are given in the supplementary data file.

## Supplementary information

Supplementary Data

Supplementary Information

Supplementary Video S1

Supplementary Video S2

Supplementary Video S3

Supplementary Video S4

Supplementary Video S5

Supplementary Video S6

Supplementary Video S7

Supplementary Video S8

## Data Availability

The Android app and source code can be accessed from our GitHub repository: https://github.com/Murawskilab/SmartphoneOptogenetics. The research data underpinning this work is available as part of the Supplementary Information.
