# Supplementary material for: Controlling the behaviour of Drosophila melanogaster via smartphone optogenetics
Source: Sci Rep. 2020 Oct 19;10:17614. doi: 10.1038/s41598-020-74448-4 (PMC7572528; doi:10.1038/s41598-020-74448-4)
Supplement: Supplementary file 2 — Supplementary Information [file 41598_2020_74448_MOESM2_ESM.pdf]

Supplementary Information

Controlling the behaviour of *Drosophila melanogaster* via smartphone optogenetics

Ilenia Meloni,[1] Divya Sachidanandan,[2,3] Andreas S. Thum,[4] Robert J. Kittel,[2,3] and Caroline Murawski[1*]

1 Kurt-Schwabe-Institut für Mess- und Sensortechnik Meinsberg e.V., Kurt-Schwabe-Str. 4, 04736 Waldheim, Germany

2 Department of Animal Physiology, Institute of Biology, Universität Leipzig, Talstr. 33, 04103 Leipzig, Germany

3 Carl-Ludwig-Institute for Physiology, Leipzig University, Liebigstraße 27, 04103 Leipzig, Germany

4 Department of Genetics, Institute of Biology, Universität Leipzig, Talstr. 33, 04103 Leipzig, Germany

* Corresponding author: caroline.murawski@ksi-meinsberg.de

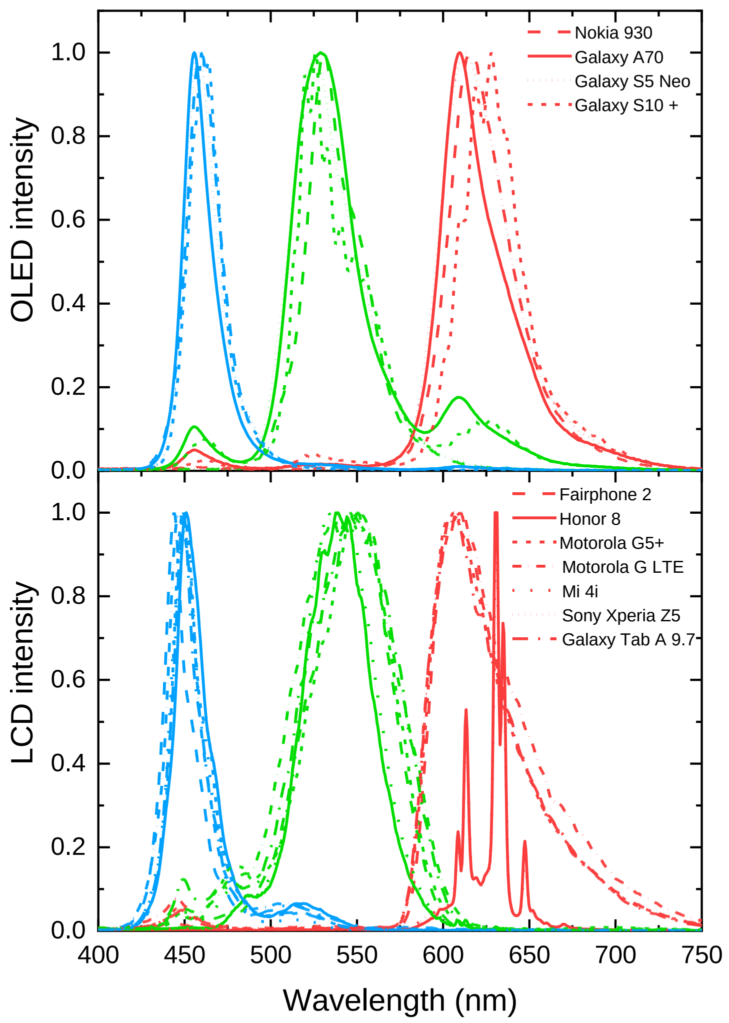

Figure S1. Emission spectra of selected OLED (top) and LCD (bottom) smartphone displays. Curves display measurements when turning on individual colours, given by RGB values: (0,0,255; blue), (0,255,0; green), and (255,0,0; red).

Table S1. Characteristics of different smartphone and tablet displays including power density and pixel area of each sub-pixel (RGB), total number of pixels, resolution, display technology, and thickness of the cover glass.

Smartphone	Red		Green		Blue		White	Pixels	Resolution (ppi)	Technology	Glass thickness (mm)
	Power density (μW/mm²)	Area (mm²)	Power density (μW/mm²)	Area (mm²)	Power density (μW/mm²)	Area (mm²)	Power density (μW/mm²)				
Fairphone 2	1.3	$6.0 * 10^{-4}$	1.4	$6.0 * 10^{-4}$	0.8	$6.0 * 10^{-4}$	3.6	1920 × 1080	446	LCD	0.8
Honor 8	1.8	$5.0 * 10^{-4}$	2.1	$5.0 * 10^{-4}$	2.1	$5.0 * 10^{-4}$	6.6	1920 × 1080	424	LCD	0.8
Motorola G5+	1.3	$1.0 * 10^{-3}$	2.0	$1.0 * 10^{-3}$	1.8	$1.0 * 10^{-3}$	5.8	1920 × 1080	441	LCD	0.9
Motorola Moto G LTE	1.5	$1.8 * 10^{-3}$	2,1	$1.8 * 10^{-3}$	1.5	$1.8 * 10^{-3}$	4.9	1280 × 720	294	LCD	0.7
Nokia Lumia 930	1.2	$5.0 * 10^{-3}$	1.4	$2.8 * 10^{-3}$	1.0	$4.0 * 10^{-3}$	4.0	1920 × 1080	441	OLED	1.2
Samsung Galaxy A70	0.9	$1.5 * 10^{-3}$	1.8	$7.0 * 10^{-4}$	1.0	$1.0 * 10^{-3}$	3.6	2400 × 1080	393	OLED	0.7
Samsung Galaxy S10+	1.8	$5.0 * 10^{-4}$	2.3	$3.0 * 10^{-4}$	1.4	$4.0 * 10^{-4}$	5.9	3040 × 1440	522	OLED	0.6
Samsung Galaxy S5 Neo	1.8	$5.0 * 10^{-4}$	1.8	$4.0 * 10^{-4}$	1.7	$7.0 * 10^{-4}$	4.2	1920 × 1080	306	OLED	0.9
Samsung Galaxy Tab A 9.7 **(Tablet)**	1.3	$1.3 * 10^{-2}$	1.9	$1.3 * 10^{-2}$	1.6	$1.3 * 10^{-2}$	5.0	1024 × 768	132	LCD	1.3
Sony Xperia Z5	1.9	$1.0 * 10^{-3}$	2.8	$1.0 * 10^{-3}$	2.8	$1.0 * 10^{-3}$	7.1	1880 × 1040	424	LCD	0.8
Xiaomi Mi 4i	0.9	$5.0 * 10^{-3}$	1.4	$5.0 * 10^{-3}$	1.3	$5.0 * 10^{-3}$	3.2	1880 × 1040	441	LCD	0.9

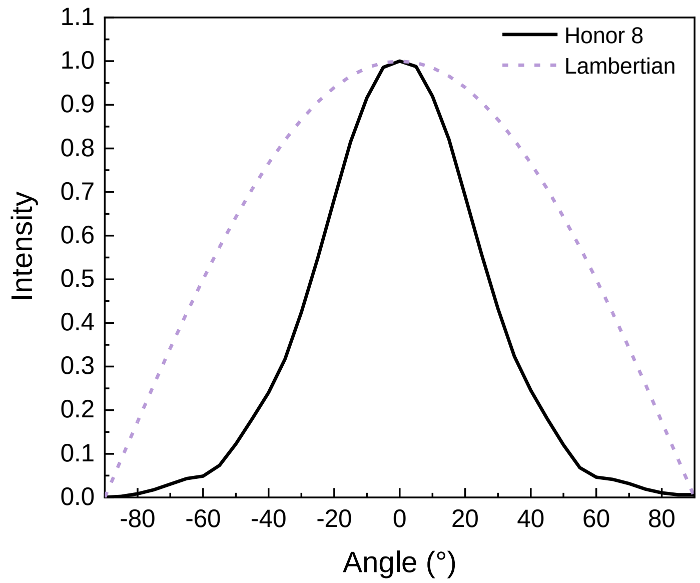

Figure S2. Angular light intensity distribution of Honor 8 display measured using a goniometer (black solid line) in comparison to Lambertian emission (purple dashed line).

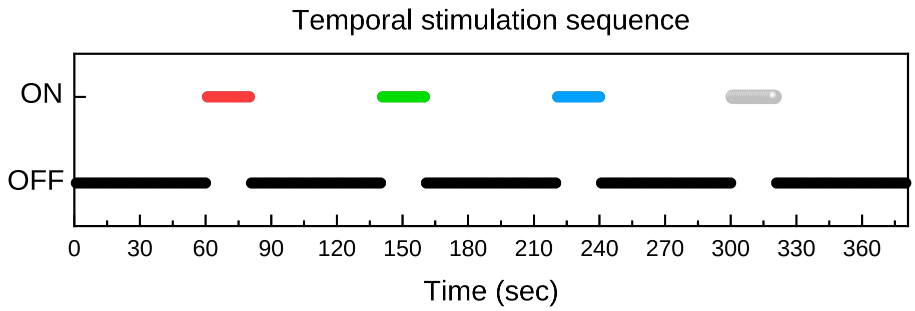

Figure S3. Sketch of an exemplary stimulation sequence used for optogenetic stimulation of *OK6 > CsChrimson* larvae. Stimulation sequence of red, green, blue, and white light for a period of 20 s with 60 s of rest (black period) between each stimulation colour.

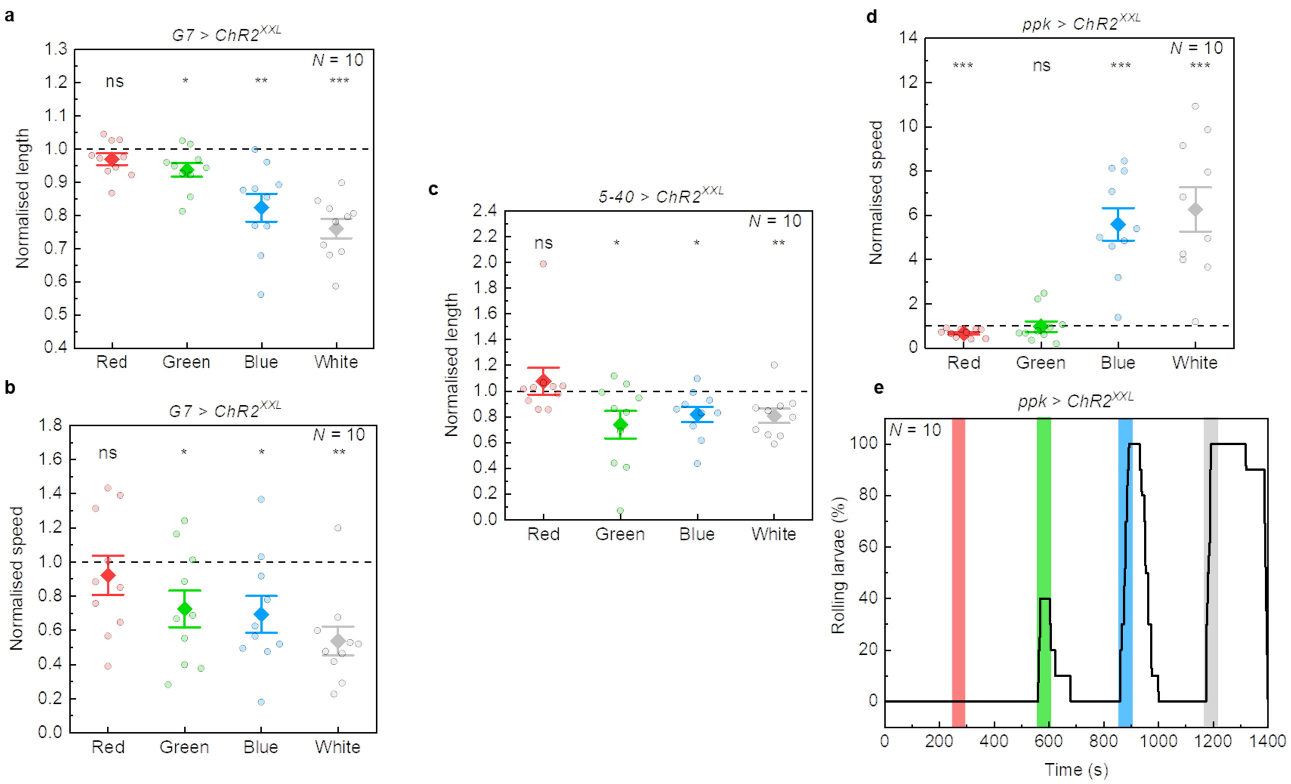

Figure S4. Optogenetic activation of different sets of neurons under expression of ChR2^{XXL}. a) Normalised length and b) Normalised speed of larvae expressing *G7 > ChR2^{XXL}*. Stimulation period: 20 s; black period: 250 s. c) Normalised length of larvae expressing *5-40 > ChR2^{XXL}*. Stimulation period: 20 s; black period: 240 s. d-e) Response of *ppk > ChR2^{XXL}* larvae upon optogenetic activation. d) Normalised speed and e) percentage of rolling larvae over time. Stimulation period: 20 s; black period: 240 s. *N*: number of larvae; whiskers: s.e.m.; diamonds: mean. Significance calculated via one-sample two-tailed t-test: ns: not significant ($p > 0.05$), * $p < 0.05$, ** $p < 0.01$, *** $p < 0.001$.

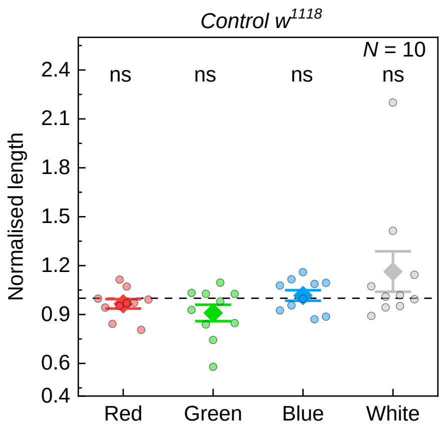

Figure S5. Normalised length of w^{1118} control larvae upon optogenetic stimulation. Stimulation period: 20 s; black period: 30 s. *N*: number of larvae; whiskers: s.e.m.; diamonds: mean. Significance calculated via one-sample two-tailed t-test: ns: not significant ($p > 0.05$).

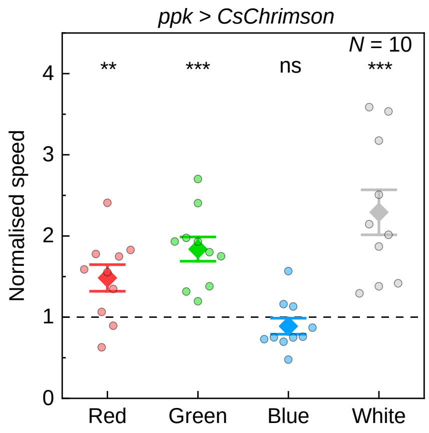

Figure S6. Normalised speed of larvae expressing *ppk > CsChrimson* upon optogenetic stimulation. Stimulation period: 20 s; black period: 40 s. *N*: number of larvae; whiskers: s.e.m.; diamonds: mean. Significance calculated via one-sample two-tailed t-test: ns: not significant ($p > 0.05$), ** $p < 0.01$, *** $p < 0.001$.

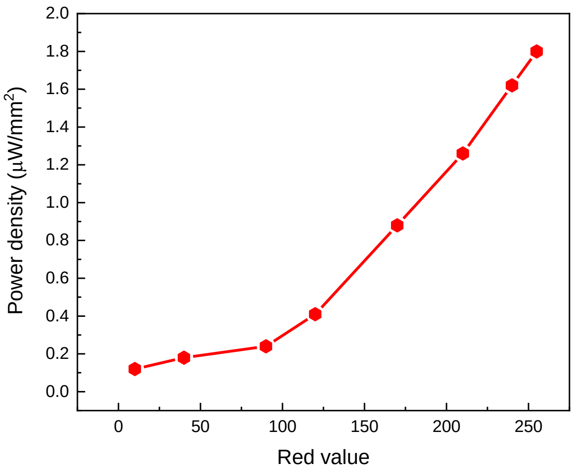

Figure S7. Power density of the Honor 8 smartphone display as a function of the set RGB value for red emission from (0,0,0) to (255,0,0).

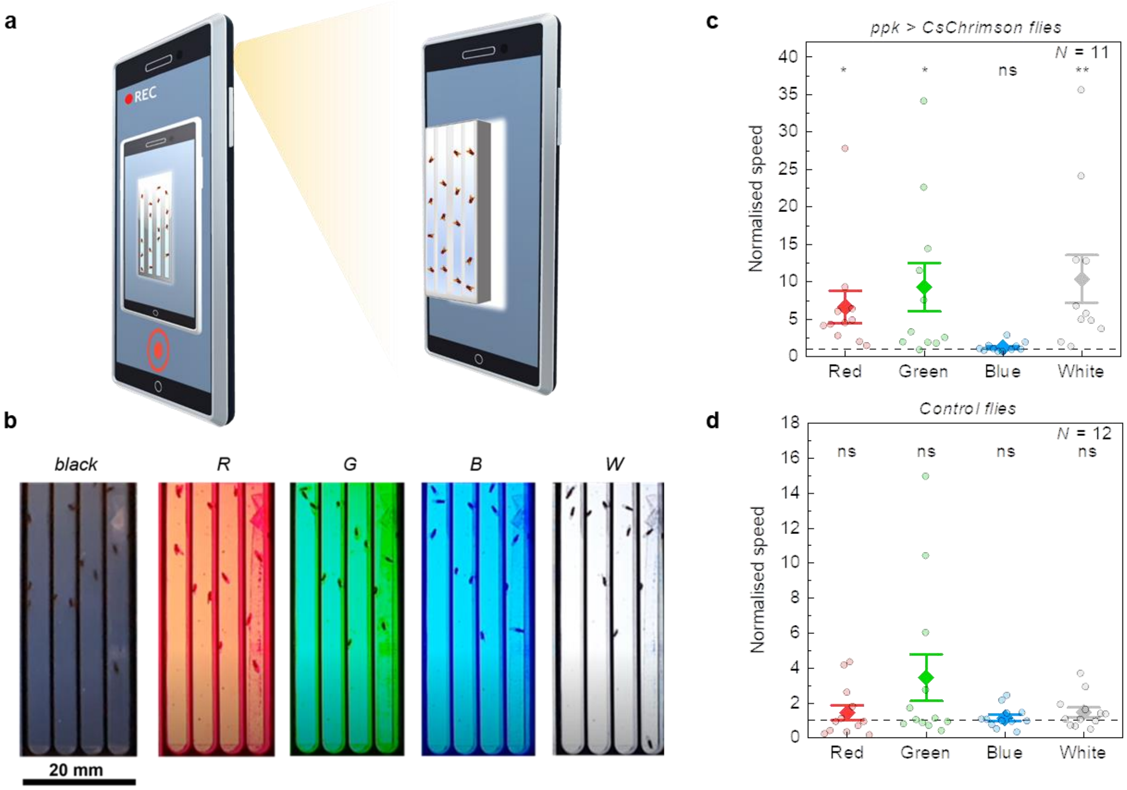

Figure S8. Control of behaviour in adult *Drosophila* with smartphone illumination. a) Sketch of the experimental setup used for stimulation of flies, which includes two smartphones (one for optogenetic stimulation and one for recording) and a chamber for containment of flies. b) Photographs of flies expressing *ppk > CsChrimson* during black periods and when stimulated with red, green, blue, and white light. c) Normalised speed of flies expressing *ppk > CsChrimson* in response to an illumination sequence with different colours for 30 s each with intermediate black periods of 40 s. Significant speed increase is observed in response to stimulation with red, green, and white light. d) Normalised speed of w^{1118} control flies in response to illumination with different colours. Illumination period: 30 s; black period: 40 s. *N*: number of flies; whiskers: s.e.m.; diamonds: mean. Significance calculated via one-sample two-tailed t-test: ns: not significant ($p > 0.05$), * $p < 0.05$, ** $p < 0.01$.

Video S1. Inhibition of motor neurons in larvae expressing *OK6 > GtACR1* using white light.

Video S2. Activation of muscles in larvae expressing *G7 >* $ChR2^{XXL}$ with white light.

Video S3. Activation of class IV multidendritic neurons in larvae expressing *ppk >* $ChR2^{XXL}$ with white light. Video accelerated to 3× speed.

Video S4. Guidance of a free-behaving *ppk > CsChrimson* larva using a ring of white light that moves at a speed of 0.17 mm/s in *x*-direction. Video accelerated to 25× speed. The red line indicates the track of the larval head.

Video S5. Observation of a free-behaving w^{1118} control larva using a ring of white light that moves at a speed of 0.17 mm/s in *x*-direction. Video accelerated to 25× speed. The red line indicates the track of the larval head.

Video S6. Confinement of 11 *ppk > CsChrimson* larvae inside a black maze surrounded by white light. Video accelerated to 125× speed. Coloured lines show tracks of the larvae.

Video S7. Video recording of 10 w^{1118} control larvae inside a black maze surrounded by white light. Video accelerated to 125× speed. Coloured lines show tracks of the larvae.

Video S8. Climbing assay of w^{1118} control flies (left) and *ppk > CsChrimson* flies (right) stimulated with white light. Video accelerated to 3× speed.
